# Efficient Intelligence with Applications in Embedded Sensing

**DOI:** 10.3390/s23104816

**Published:** 2023-05-17

**Authors:** Xingxing Zuo, Yong Liu

**Affiliations:** 1 School of Computation, Information and Technology, Technical University of Munich, 85748 Garching, Germany; xingxing.zuo@tum.de; 2 Institute of Cyber-Systems and Control, Zhejiang University, Hangzhou 310027, China

## 1. Introduction

Despite the fact that computational technology continues to rapidly develop, edge devices and embedded systems are still limited in terms of their computation resources due to such factors as power consumption, physical size constraints, and manufacturing cost. This poses a challenge for critical applications such as mobile robots, cell phones, and AR and VR devices, which require efficient sensing with sensors and on-board computational resources. To effectively process the abundance of sensor measurements using resource-constrained computation platforms, there is a need to limit the computation complexity of the methods deployed. This is true whether the method is data-driven or principle-driven, and high efficiency is typically a critical requirement.

This Special Issue is focused on both practical and theoretical technologies in the field of efficient intelligence and how they can be applied to diverse embedded devices such as industrial robots, unmanned vehicles, and fuel cells. The ten research papers published in this Special Issue cover a wide range of topics, including collaborative autonomous navigation with unmanned surface and aerial vehicles, multi-modal simultaneous localization and mapping (SLAM), target object tracking, LiDAR point cloud loop closure detection, motion distortion compensation for LiDAR point cloud, hybrid prognostic methods for proton-exchange-membrane fuel cells (PEMFC), detection of fabric defects during factory manufacturing, state recognition of elevator traction machines, efficient object detection neural networks, accurate pantograph detection for high-speed railways, and vision-based autonomous forklifts. It is our hope that these published papers will be beneficial for both academic researchers and relevant industrial practitioners alike.

## 2. Overview of Contribution

To ensure that an unmanned surface vehicle (USV) can navigate safely in complex scenarios with many obstacles, Huang et al. [1] proposed a system that involves multiple agents collaborating together. This system includes an unmanned aerial vehicle (UAV) that acts as a perceptive agent with a large receptive field, allowing it to detect obstacles from above and inform the USV of their locations. Next, a graph search-based hybrid A* planning algorithm generates an obstacle-free trajectory for the USV. This initial trajectory is further optimized by taking into account the dynamic constraints of the under-actuated USV. By doing so, the planned trajectory is tailored to the USV’s dynamics, making it easier for the vehicle to follow. Finally, a nonlinear model predictive controller (NMPC) with the lowest energy consumption constraint is used to control the USV and ensure it follows the planned trajectory precisely. The effectiveness and efficiency of this collaborative system have been demonstrated in a simulated environment.

Chen et al. [2] proposed a heterogenous Simultaneous Localization and Mapping (SLAM) system that combines sensor measurements from LiDAR, cameras, Inertial Measurement Units (IMU), and Global Positioning Systems (GPS). This system has three component state estimation subsystems: LiDAR-inertial odometry, visual-inertial odometry, and GPS-inertial odometry. The navigation states estimated from these subsystems are then fused in a pose graph optimization. This heterogenous hybrid SLAM system is designed to provide accurate and robust pose estimations, even in complex environments and difficult situations, such as when there are component sensor failures or intermittent GPS measurements. Additionally, based on the estimated camera poses from the SLAM system, an object tracking and localization module has been developed. This object tracking and localization module utilizes YOLOv4 to detect objects of interest from images captured by the camera. The detected objects are then tracked across multiple images using L-K optical flow and a Kalman filter. With the known 6-DoF relative transformation between the camera and LiDAR, the depth of the object can be obtained by the projected LiDAR points. This allows for the retrieval of 3D locations of the tracked objects. Experimental results in real-world scenarios have demonstrated the accurate pose estimation of the sensor rig, as well as the feasibility of tracking object locations using the presented multi-modal system.

When using odometry systems, there will inevitably be a slight drift in the estimation of the robot’s position. This is because minor errors in the relative poses between consecutive frames accumulate over time, leading to significant deviations in the long run. To fix this problem, loop closures can be used. When the robot reaches a previously visited position, a loop closure is triggered, and the accumulated drift during that loop can be corrected. Tian et al. [3] proposed a method to improve loop closure detection in 3D LiDAR scans by using an object segmentation technique. The method uses a Scan Context descriptor, which is a global descriptor that records statistics of the 3D structure captured by LiDAR and stores the descriptors in an indexed KD-Tree. Loop closure candidates are identified as scans with small descriptor distance to existing scans. To enhance the performance of loop closure detection in complex environments, the method uses object segmentation to remove disturbances caused by unstructured objects such as cluttered vegetation. Experimental results on the KITTI dataset demonstrate that the proposed method outperforms the other compared methods.

To provide a 360-degree panoramic perception of the environment, mechanical 3D LiDARs use spinning laser sensors. However, if the LiDAR is moving during the spinning of the laser sensors, motion distortion can occur in the LiDAR scan. To solve this problem, Wu et al. [4] proposed a method that fuses IMU and wheel odometer measurements to compensate for the motion distortion in LiDAR scans. The positional displacement from the wheel odometer measurements and rotation changes from the IMU measurements are combined to estimate the 6 degrees of freedom (DoF) pose of the LiDAR. To roughly remove the motion distortion of a LiDAR scan, the pose estimations are linearly interpolated to obtain the interpolated poses for individual LiDAR points. Then, the roughly undistorted LiDAR scans are registered with each other via ICP (Iterative Closest Point), and the relative poses between the LiDAR scans are calculated. The relative poses obtained from the registration process are used to further undistort the motion distortion in LiDAR scans. Extensive experiments have shown that this proposed method is effective and feasible in compensating for motion distortion in LiDAR scans.

Xia et al. [5] proposed a new method for predicting the long-term voltage degradation of proton-exchange-membrane fuel cells (PEMFC) using a hybrid prognostic approach. The voltage measured from the PEMFC is decomposed into two components: the calendar aging component and the reversible aging component. To predict the overall aging trend of the PEMFC based on the calendar aging component, an adaptive extended Kalman filter is used. Additionally, a Long Short-Term Memory (LSTM) neural network is utilized to predict both voltage components together. The combination of the Kalman filter and LSTM helps to accurately predict the long-term voltage degradation of PEMFC. Furthermore, to improve the accuracy of the forecast, a dedicated three-dimensional aging factor is introduced into the physical aging model. Experimental results show that the proposed hybrid prognostic method delivers accurate long-term voltage-degradation prediction results, demonstrating its effectiveness over other methods.

Detecting fabric defects during factory manufacturing is crucial for ensuring high-quality products. Lin et al. [6] proposed an intelligent and efficient method for detecting fabric defects based on the YOLOv5 neural network. To overcome the challenges of detecting small and unbalanced defect patches, they modified the baseline YOLOv5 network using the Swin transformer backbone. They also incorporated a sliding-window multi-head self-attention mechanism to enhance accuracy in addition to the convolutional neural network. Furthermore, to improve detection accuracy, even on small defects, they introduced a detection layer capable of detecting 4 × 4 small targets, enabling detection at four different scales. To address the issue of imbalanced training samples, they used a generalized focal loss to help the model learn from positive samples. The proposed neural network was rigorously tested through ablation studies to analyze the effectiveness of each introduced network component. The experimental results demonstrate the high detection accuracy and real-time capability of the proposed neural network, making it a useful tool for fabric defect detection in factory manufacturing.

The condition monitoring and fault diagnosis of elevator traction machines is incredibly important for ensuring the safety of elevator users. Li et al. [7] proposed a new method for recognizing the state of a traction machine based on analyzing its vibration signals. To do this, they use a novel demodulation method that involves time-frequency analysis and principal component analysis. In order to extract the important modulation characteristics of the traction machine vibration signal, which can be difficult due to background noise interference, the researchers employ two methods: Fast Fourier Transform (FFT) and Short-Time Fourier Transform (STFT). They conduct extensive investigations while the elevator runs at different speeds, in different directions and with varying weights. The results show that applying principal component analysis is very helpful for quickly and effectively monitoring the condition of a traction machine in different scenarios. Overall, this method can help ensure that elevator traction machines are operating safely.

Yun et al. [8] proposed an effective vision-based method for recognizing objects that involves two main stages. In the first stage, a lightweight semantic segmentation neural network called ENet is used to extract the Region of Interest (ROI). This allows the objects that are not of interest or that are part of the background to be masked out. The areas that have the potential to contain objects of interest are the only regions that will be recognized. In the second stage, the masked image from the first stage is processed through the YOLO neural network to achieve efficient and accurate recognition. While the results from the first stage may not be perfect, the second stage can still achieve high accuracy. Experiments on embedded devices have demonstrated that using this two-stage method for object recognition not only saves power and computation but also significantly improves accuracy.

High-speed trains rely on a pantograph to provide power by connecting to the powerlines. The pantograph’s status is critical to the functioning of the high-speed railway (HSR). To detect and locate the pantograph in images captured by a specific camera, Tan et al. [9] developed a dedicated detection method that uses YOLOv4. They trained this model on data collected from real-world scenarios. Since the camera that watches the pantograph is mounted outside of the train, it can be susceptible to various types of interference, such as rainwater-induced blurring or dirt on the camera lens. To better understand the health status of the camera and analyze the interference affecting the performance of YOLOv4 detection, a classification method is proposed. This method counts the number of blobs appearing in the image to determine if the camera is affected by dirt or blur. In addition, since the image backgrounds of the photograph can be diverse in different scenarios and can significantly affect the detection performance of YOLOv4, a method was developed to infer the categories of complex backgrounds. Overall, this proposed system provides an effective and efficient way to detect and locate the pantograph on high-speed trains, despite the challenges posed by environmental factors.

Ren et al. [10] proposed a complete system that enables forklifts to transfer pallets accurately and efficiently in warehouse environments. This system has three main components: pallet monitoring using an RGB surveillance camera, pallet positioning with an RGB-D camera mounted on the forklift, and a dedicated controller that instructs the forklift to manipulate the pallet with high precision. To detect pallets that are far away from the cameras in the pallet monitoring module, a transformer-based prediction head is incorporated into the YOLOv5 network. This allows for the detection of small targets that correspond to only a few pixels. For pallet positioning, deep feature maps are generated from the RGB-D images and fed into a 3D key points detection network. This network accurately detects the eight corner points of the pallets’ two square apertures. By fitting the extracted key points, the pose of the pallet relative to the forklift can be determined. Once the pose of a pallet is known, the forklift is controlled to transfer the pallet with a trajectory controller that incorporates forklift motion cycle prediction into the control process. The proposed system has been extensively tested in real-world warehouse scenarios and has been shown to be effective and reliable.

## 3. Conclusions

This Special Issue encompasses a diverse array of topics related to efficient sensing and intelligence for embedded devices. The advancements showcased in the ten research papers published within this Special Issue highlight the significance of devising practical and theoretical technologies to tackle challenges posed by resource-constrained computation platforms and adverse external interference across various applications, including robotics, manufacturing, transportation, and energy systems. These cutting-edge solutions strive to enhance the efficiency, accuracy, and robustness of embedded sensing and intelligence while surmounting physical limitations and diverse environmental obstacles. The research papers in this collection contribute to the progress of efficient intelligence technologies, offering valuable insights and inspiration for both academic researchers and industry practitioners in their quest to develop more advanced and efficient embedded sensing systems.
